# Endoscopic resection for a solitary Peutz‐Jeghers type polyp in the duodenum: A case report with literature review

**DOI:** 10.1002/deo2.226

**Published:** 2023-03-27

**Authors:** Yasuhiko Hamada, Masaki Katsurahara, Yuhei Umeda, Yohei Ikenoyama, Akina Shigefuku, Yasuko Fujiwara, Tuyoshi Beppu, Junya Tsuboi, Reiko Yamada, Misaki Nakamura, Kyosuke Tanaka, Noriyuki Horiki, Hayato Nakagawa

**Affiliations:** ^1^ Department of Gastroenterology and Hepatology Mie University Hospital Tsu Mie Japan

**Keywords:** duodenum, endoscopic resection, hamartomatous polyp, Peutz‐Jeghers syndrome, Peutz‐Jeghers‐type polyp

## Abstract

A 68‐year‐old female patient was referred to our hospital with a 30‐mm polyp in the second portion of the duodenum found via esophagogastroduodenoscopy. The polyp had an irregular, lobular surface and a thick stalk. In addition, white dots were detected on the surface. Magnifying endoscopy with narrow‐band imaging showed a white material deep in the loop‐shaped microvessels on the white dots. Endoscopic ultrasonography showed a hypoechoic elevated lesion from the mucosal layer, and a feeding vessel traversing the stalk to supply the head of the polyp. Endoscopic biopsy did not provide a definitive diagnosis. Endoscopic resection was conducted for a definitive diagnosis and treatment. The resected specimen showed a branching bundle of smooth muscle fibers covered by hyperplastic mucosa, consistent with a hamartomatous polyp. The patient had no mucocutaneous pigmentation or familial history of the hamartomatous polyp. The polyp was finally diagnosed as a solitary Peutz‐Jeghers‐type polyp. No recurrence has been observed for seven years postoperatively.

## INTRODUCTION

Peutz‐Jeghers (PJ) syndrome is a rare autosomal dominant syndrome caused by mutations in the serine/threonine kinase 11 genes, which is characterized by gastrointestinal hamartomatous polyps, mucocutaneous pigmentation, and an increased risk of cancer.^1^, ^2^, ^3^

A hamartomatous polyp without associated mucocutaneous pigmentation or a family history of PJ syndrome is diagnosed as a solitary PJ‐type polyp.^4^ A solitary PJ‐type hamartomatous polyp is histologically characterized by tree‐like branching of smooth muscle fibers, with a smooth muscle core, covered by mucosal tissue of near‐normal appearance.^5^ This type of hamartomatous polyp is now considered a separate disease entity from that of PJ syndrome. However, a solitary PJ‐type polyp in the duodenum is rare, and determining the definitive diagnosis is challenging due to the absence of specific clinical findings and the difficulty of differentiating it from neoplastic lesions.

This case report discusses a patient with a solitary PJ‐type polyp in the duodenum treated by endoscopic resection and includes a review of the literature.

## CASE REPORT

**FIGURE 1:**
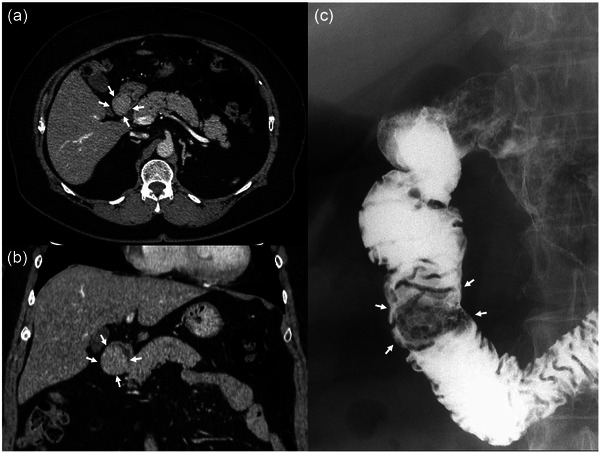
Computed tomography and duodenography findings. Enhanced computed tomography image shows a homogeneous, well‐circumscribed mass with a diameter of 30 mm in the second portion of the duodenum (a: axial image; b: coronal image; arrows). (c) A duodenography reveals a 30‐mm well‐defined mass in the second portion of the duodenum (arrows).

**FIGURE 2:**
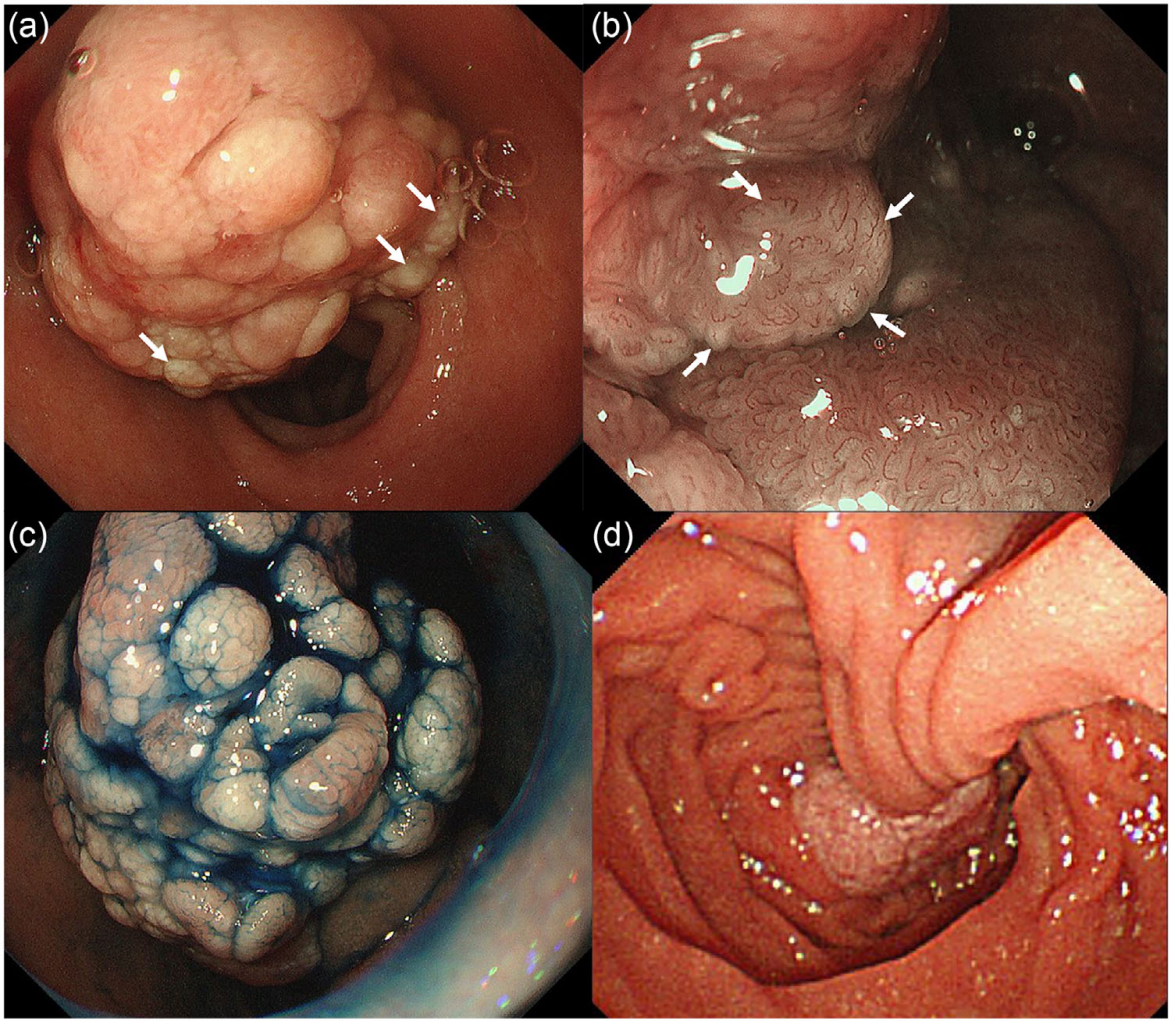
Esophagogastroduodenoscopy findings. (a) Esophagogastroduodenoscopy reveals a 30‐mm, pedunculated polyp originating in the second portion of the duodenum. The polyp has white dots on the surface (arrows). (b) Magnifying endoscopy with narrow‐band imaging showed a white material deep in the loop‐shaped microvessels on the white dots (arrows). (c) Chromoendoscopy with indigo carmine dye reveals an irregular, lobular surface. (d) Side‐viewing endoscopy shows that the polyp has a thick stalk.

**FIGURE 3:**
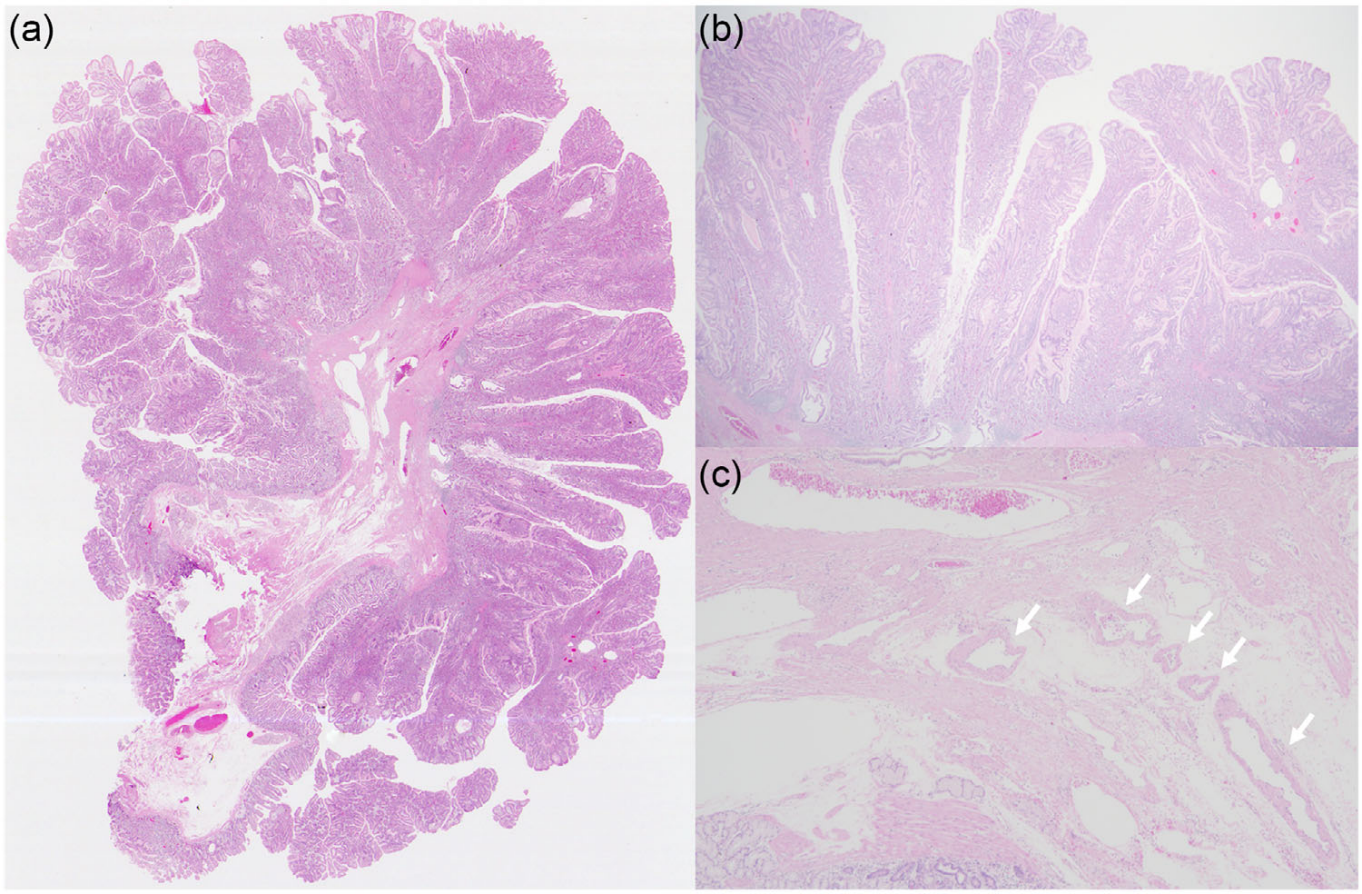
Pathology (hematoxylin and eosin stain). (a) Low‐power view of the resected specimen shows a cauliflower‐like polypoid lesion consisting of branching bundles of smooth muscle fibers. (b) Several cystic glands are seen in the epithelium but there was no dysplasia (magnification: ×12.5). (c) Feeding vessels traversing the stalk were observed inside the stalk (magnification: ×40, arrows).

A 68‐year‐old female patient with a history of hypertension and diabetes mellitus was referred to our hospital with a pedunculated polyp in the duodenum identified by routine esophagogastroduodenoscopy. Physical examination and blood tests revealed no significant findings. Contrast‐enhanced computed tomography revealed a well‐circumscribed mass in the 2nd portion of the duodenum (Figure 1a: axial image; b: coronal image; arrows). A duodenography revealed a well‐defined mass, measuring approximately 30 mm in size, in the 2nd portion of the duodenum (Figure 1c; arrows). Esophagogastroduodenoscopy revealed the polyp to be 30 mm with white dots on the surface (Figure 2a, arrows). On the white dots, magnifying endoscopy with narrow‐band imaging showed a white material deep in the loop‐shaped microvessels (Figure 2b, arrows). Chromoendoscopy with indigo carmine dye revealed an irregular, lobular surface (Figure 2c), and side‐viewing endoscopy revealed that the polyp had a thick stalk (Figure 2d). Endoscopic ultrasonography showed a hypoechoic elevated lesion from the mucosal layer, and a luminal‐like structure was observed inside the stalk (Figure S1a, arrows). Color Doppler imaging showed a blood flow inside the luminal‐like structure; accordingly, it was considered a feeding vessel traversing the stalk to supply the head of the polyp (Fig. S1b, arrows). Based on the endoscopic appearance, the differential diagnoses considered included PJ‐type polyp, adenoma, and cancer. However, biopsy specimens obtained from the polyp revealed a hyperplastic duodenal mucosa. After a discussion with the patient, endoscopic mucosal resection was performed for a definite diagnosis and treatment (Figure S2a–d). In the endoscopic resection, prophylactic hemostatic clip applications to the base of the stalk were attempted before endoscopic snare resection; however, it failed because of poor endoscope manipulation. Thus, the endoscopic resection was performed using coagulation mode to prevent preoperative bleeding. Pathological examination of the resected specimen revealed a branching bundle of smooth muscle fibers covered by hyperplastic duodenal mucosa, consistent with a hamartomatous polyp (Figure 3a,b). A feeding vessel detected by the endoscopic ultrasonography was pathologically confirmed in the resected specimen (Figure 3c, arrows). No hamartomatous polyps in the colon or small intestine were detected via colonoscopy or video capsule endoscopy, respectively. Therefore, the duodenal polyp was finally diagnosed as a solitary PJ‐type polyp in the duodenum. Postoperatively, the patient recovered well, with no recurrence for seven years after the treatment.

## DISCUSSION

A previous case series that included 51 patients with a solitary PJ‐type gastrointestinal polyp reported that the sigmoid colon was most frequently involved, followed by the rectum, duodenum, transverse colon, jejunum, and cecum.^6^ Overall, a solitary PJ‐type polyp was found in the large intestine in 39 patients (77%) and the duodenum in 10 patients (20%). PJ‐type polyps may be differentiated from PJ syndrome. Symptom onset typically occurs in the sixth and seventh decades of life and is associated with a lower risk of neoplasia development than hamartomatous polyps in patients with PJ syndrome.

A MEDLINE search of the well‐documented English language literature up to 2022 using the terms “hamartomatous polyp” and “duodenum” revealed 15 studies (including the current study), including 21 patients with a solitary PJ‐type polyp in the duodenum (Table 1: the reference articles are described in Doc S1). The mean age at diagnosis was 58.8 years (range 23–87 years). The most frequent polyp location was the second portion of the duodenum, followed by the duodenal bulb. The mean polyp diameter is 27.5 mm (range 5–70). Among the 17 cases with well‐documented polyp morphology, the most frequent polyp morphology was a pedunculated type (14/17, 82.4%). More than half of the well‐documented 20 cases were symptomatic (13/20, 65%), which differs from the cases with superficial non‐ampullary duodenal tumors that were usually asymptomatic. Most lesions underwent endoscopic resection, although three patients with large lesions (>50 mm) required surgical resection. Of the 21 lesions, five had focal dysplasia.

**TABLE 1:** Case series of solitary Peutz‐Jeghers‐type polyp in the duodenum (English literature only).

**Case no**.*	**Author**	**Publish year**	**Age (years)**	**Sex**	**Clinical symptoms**	**Polyp location**	**Polyp size (mm)**	**Morphology**	**Biopsy result**	**Treatment**	**Dysplasia**
1	Bott et al.	1986	23	Male	GI bleeding	4th	50	ND	Inflammation	Surgical resection	No
2	Naitoh et al.	1988	56	Female	ND	3rd	30	Pedunculated	ND	Endoscopic resection	No
3	Tanaka et al.	1990	41	Male	Asymptomatic	3rd	25	Pedunculated	Non‐neoplastic	Endoscopic resection	No
4	‐	‐	82	Female	Asymptomatic	2nd	25	Pedunculated	Adenoma	Endoscopic resection	No
5	Acea Nebri et al.	1993	63	Female	Anemia	1st	50	ND	Hyperplastic	Surgical resection	No
6	Ichiyoshi et al.	1996	84	Female	GI bleeding	2nd	25	Pedunculated	Non‐neoplastic	Endoscopic resection	Yes
7	Oncel et al.	2003	68	Female	GI bleeding	3rd	25	Pedunculated	ND	Endoscopic resection	No
8	‐	‐	53	Male	Dyspepsia	2nd	5	ND	ND	Endoscopic resection	No
9	Kitaoka et al.	2004	22	Female	Asymptomatic	1st	30	ND	ND	Endoscopic resection	No
10	Itaba et al.	2006	87	Female	Abdominal pain	2nd	18	Pedunculated	Normal	Endoscopic resection	No
11	‐	‐	56	Male	Tumor marker	2nd	12	Pedunculated	Normal	Endoscopic resection	No
12	Suzuki et al.	2008	59	Female	Abdominal pain	2nd	15	Semi‐pedunculated	Hyperplastic	Endoscopic resection	No
13	‐	‐	68	Female	Abdominal pain	2nd	10	Pedunculated	Adenoma	Endoscopic resection	Yes
14	‐	‐	60	Female	Asymptomatic	1st	10	Semi‐pedunculated	Hyperplastic	Endoscopic resection	No
15	Jamaludin et al.	2009	46	Male	Dyspepsia	1st	70	Pedunculated	Adenocarcinoma	Surgical resection	Yes
16	Kantarciogle et al.	2009	28	Female	Melena	2nd	25	Pedunculated	ND	Endoscopic resection	No
17	Sekino et al.	2011	84	Male	Pancreatitis	1st	14	Pedunculated	ND	Endoscopic resection	Yes
18	‐	‐	76	Male	Asymptomatic	2nd	15	Pedunculated	ND	Endoscopic resection	No
19	Suzuki et al.	2015	89	Male	Cholangitis	2nd	48	Pedunculated	ND	Endoscopic resection	No
20	Rathi et al.	2016	22	Male	Asymptomatic	2nd	45	Sessile	Adenoma	Endoscopic resection	Yes
21	Our case	2023	68	Female	Asymptomatic	2nd	30	Pedunculated	Hyperplastic	Endoscopic resection	No

Abbreviations: GI, gastrointestinal; ND, not described.

^*^Reference articles are described in Doc S1.

The differential diagnosis based on morphological features is difficult as PJ‐type polyps present with various colors, including normal, erythematous, and white, and often present with pedunculated or semi‐pedunculated morphology, which is similar to duodenal neoplastic lesions. In addition, differentiation of PJ‐type polyp from neoplastic lesions in the duodenum is difficult by endoscopic ultrasonography. Sonoda et al. reported knacks of endoscopic differentiation between PJ‐type and neoplastic polyps that included: (1) due to the branching bundles of smooth muscle fibers, PJ‐type polyps have more irregular lobules than neoplastic polyps; and (2) PJ‐type polyps have white dots, which represents lymphatic congestion rather than whitened epithelium as often seen in neoplastic polyps, which can be confirmed by narrow‐band imaging magnifying endoscopy.^7^ These endoscopic appearances were detected in this case.

To date, there is no definitive therapeutic strategy for solitary PJ‐type polyps in the duodenum. A definitive diagnosis by endoscopic biopsy is difficult as PJ‐type polyps are typically covered by the hyperplastic duodenal mucosa. Most of the 11 cases with well‐documented biopsy results in the literature were not definitively diagnosed (Table 1). Moreover, previous reports have described that solitary PJ‐type polyps may develop dysplasia, although the risk is lower compared to those associated with PJ syndrome.^8^ Therefore, if technically possible, endoscopic resection should first be conducted to achieve a definitive diagnosis and treatment for a solitary PJ‐type polyp. In long‐term outcomes after endoscopic resection, previous studies have reported no recurrence or death related to solitary PJ‐type polyps.^6^

In this case, endoscopic ultrasonography showed a large vessel inside the stalk, which was pathologically confirmed in the resected specimen. Thus, prophylactic hemostatic clip applications to the base of the stalk should be attempted before endoscopic snare resection. In our literature review, 82.4% of cases with solitary PJ‐type polyps in the duodenum were pedunculated‐type morphology. Large pedunculated polyps usually have a large feeding vessel traversing the stalk to supply the head of the polyp and present a high risk for bleeding after endoscopic resection.^9^ A randomized controlled study reported that prophylactic hemostatic clips could reduce bleeding after endoscopic resection of large pedunculated polyps.^10^ Thus, prophylactic hemostatic clip application should be attempted before endoscopic resection for the PJ‐type polyps in the duodenum.

In conclusion, we experienced a case of solitary PJ‐type polyp in the duodenum. The polyp was completely removed via endoscopic resection and no recurrence was observed. Although endoscopic findings may help to differentiate the PJ‐type polyps from neoplastic polyps, patients with a solitary PJ‐type polyp in the duodenum are recommended to undergo resection to achieve a definitive diagnosis and treatment because of the malignant potential.

## CONFLICT OF INTEREST STATEMENT

None.

## ETHICS STATEMENT

All procedures followed were performed in accordance with the ethical standards laid down in the Declaration of Helsinki and its later amendments.

## Supporting information

Figure S1 Endoscopic ultrasonography.
(a) Endoscopic ultrasonography showed a hypoechoic elevated lesion from the mucosal layer, and a luminal‐like structure was observed inside the stalk (arrows).
(b) Color Doppler imaging showed an abundant blood flow inside the luminal‐like structure (arrows).

Figure S2 Endoscopic resection.
(a) The Peutz‐Jeghers‐type polyp is shown in the second portion of the duodenum.
(b) The polyp is snared.
(c) An ulcer after endoscopic resection.
(d) The resected specimen is shown.

Doc S1 Reference articles in Table 1.
