# Supplementary figures and images for: Endoscopic resection for a solitary Peutz‐Jeghers type polyp in the duodenum: A case report with literature review
Source: DEN Open. 2023 Mar 27;3(1):e226. doi: 10.1002/deo2.226 (PMC10043356; doi:10.1002/deo2.226)

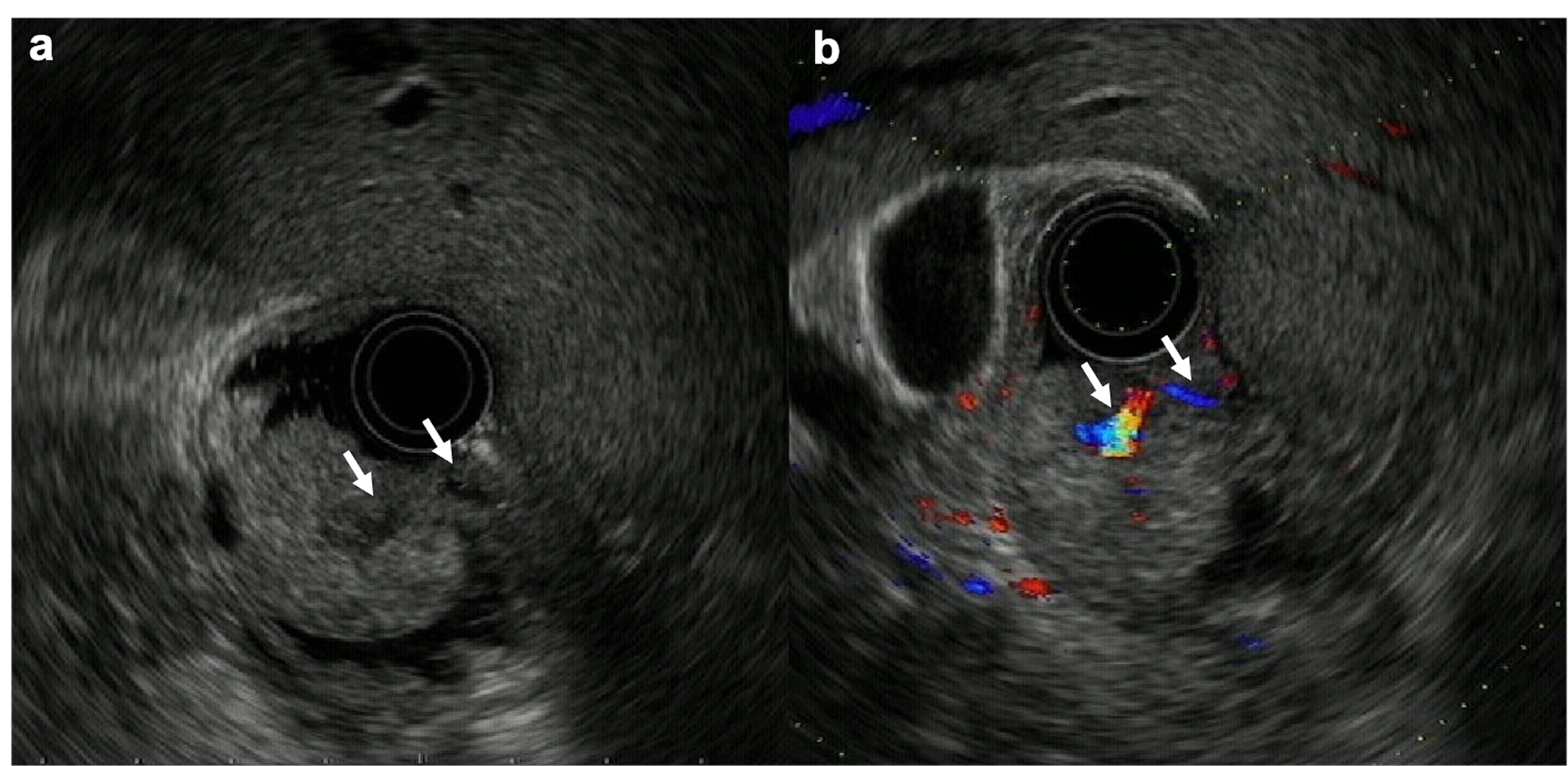

Supplement: Supplementary file 1 — Figure S1 Endoscopic ultrasonography. (a) Endoscopic ultrasonography showed a hypoechoic elevated lesion from the mucosal layer, and a luminal‐like structure was observed inside the stalk (arrows). (b) Color Doppler imaging showed an abundant blood flow inside the luminal‐like structure (arrows). [file DEO2-3-e226-s002.tiff]

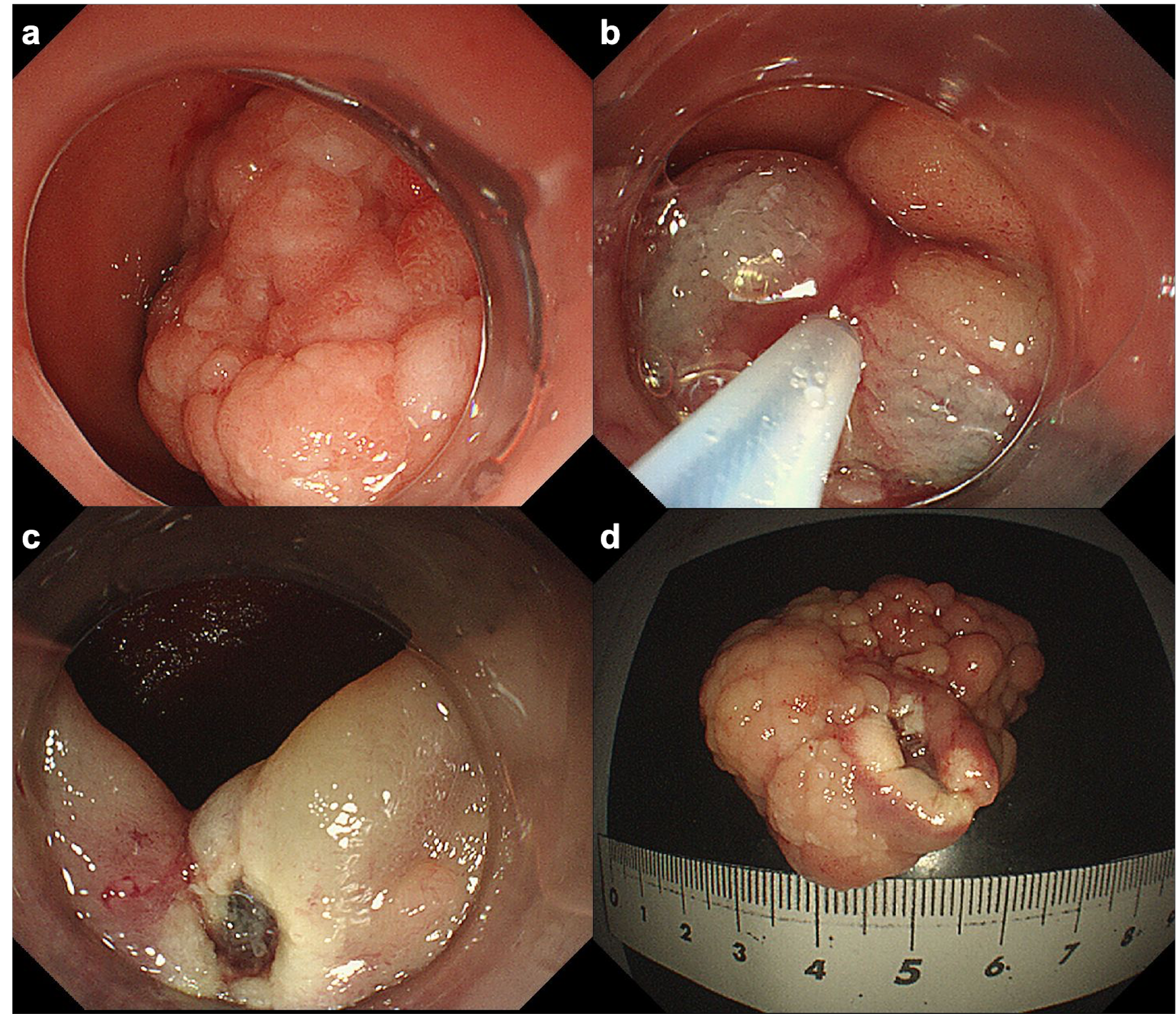

Supplement: Supplementary file 2 — Figure S2 Endoscopic resection. (a) The Peutz‐Jeghers‐type polyp is shown in the second portion of the duodenum. (b) The polyp is snared. (c) An ulcer after endoscopic resection. (d) The resected specimen is shown. [file DEO2-3-e226-s003.tiff]
