# Supplementary material for: Endoscopic resection for a solitary Peutz‐Jeghers type polyp in the duodenum: A case report with literature review
Source: DEN Open. 2023 Mar 27;3(1):e226. doi: 10.1002/deo2.226 (PMC10043356; doi:10.1002/deo2.226)
Supplement: Supplementary file 3 — Doc S1 Reference articles in Table 1. [file DEO2-3-e226-s001.docx]

**Reference articles in Table 1**

**Case no. 1**

Bott SJ, Hanks JB, Stone DD. Solitary hamartomatous polyp of the duodenum in the absence of familial polyposis. *Am J Gastroenterol*. 1986; 81(10): 993–4.

**Case no. 2**

Naitoh H, Sumiyoshi Y, Kumashiro R et al. A solitary Peutz-Jeghers type hamartomatous polyp in the duodenum--a case report. *Jpn J Surg* 1988; 18(4): 475–7.

**Case no. 3, 4**

Tanaka H, Iida M, Kohrogi N et al. Endoscopic removal of solitary hamartomatous polyps of the duodenum. Gastrointest Endosc. 1990; 36(6): 640–2.

**Case no. 5**

Acea Nebril B, Taboada Filgueira L, Parajó Calvo A et al. Solitary hamartomatous duodenal polyp; a different entity: report of a case and review of the literature. *Surg Today* 1993; 23(12): 1074–7.

**Case no. 6**

Ichiyoshi Y, Yao T, Nagasaki S, Sugimachi K. Solitary Peutz-Jeghers type polyp of the duodenum containing a focus of adenocarcinoma. *Ital J Gastroenterol* 1996; 28(2): 95–7.

**Case no. 7, 8**

Oncel M, Remzi FH, Church JM, Goldblum JR, Zutshi M, Fazio VW. Course and follow-up of solitary Peutz-Jeghers polyps: a case series. *Int J Colorectal Dis* 2003; 18(1): 33–5.

**Case no. 9**

Kitaoka F, Shiogama T, Mizutani A et al. A solitary Peutz-Jeghers-type hamartomatous polyp in the duodenum. A case report including results of mutation analysis. *Digestion* 2004; 69(2): 79–82.

**Case no. 10, 11**

Itaba S, Namoto M, Somada S et al. Two cases of solitary Peutz-Jeghers-type hamartoma of the duodenum. *Endoscopy* 2006; 38 Suppl 2: E32–3.

**Case no. 12–14**

Suzuki S, Hirasaki S, Ikeda F, Yumoto E, Yamane H, Matsubara M. Three cases of solitary

Peutz-Jeghers-type hamartomatous polyp in the duodenum. *World J Gastroenterol* 2008; 14(6): 944–7.

**Case no. 15**

Jamaludin AZ, Telisinghe PU, Yapp SK, Chong VH. Solitary duodenal hamartomatous polyp with malignant transformation: report of a case. *Surg Today* 2009; 39(6): 527–32.

**Case no. 16**

Kantarcioglu M, Kilciler G, Turan I et al. Solitary Peutz-Jeghers-type hamartomatous polyp as a cause of recurrent acute pancreatitis. *Endoscopy* 2009; 41 Suppl 2: E117–8.

**Case no. 17, 18**

Sekino Y, Inamori M, Hirai M et al. Solitary Peutz-Jeghers type hamartomatous polyps in the duodenum are not always associated with a low risk of cancer: two case reports. *J Med Case Rep* 2011; 5: 240.

**Case no. 19**

Suzuki K, Higuchi H, Shimizu S, Nakano M, Serizawa H, Morinaga S. Endoscopic snare papillectomy for a solitary Peutz-Jeghers-type polyp in the duodenum with ingrowth into the common bile duct: case report. *World J Gastroenterol* 2015; 21(26): 8215–20.

**Case no. 20**

Rathi CD, Solanke DB, Kabra NL, Ingle MA, Sawant PD. A rare case of solitary Peutz Jeghers type hamartomatous duodenal polyp with dysplasia! *J Clin Diagn Res* 2016; 10(7): Od03–4.

**Case no. 21**

Our case.
